# Prognostic Outcome of New-Onset Left Bundle Branch Block After Transcatheter Aortic Valve Replacement in Patients With Aortic Stenosis: A Systematic Review and Meta-Analysis

**DOI:** 10.3389/fcvm.2022.842929

**Published:** 2022-04-08

**Authors:** Jialu Wang, Shidong Liu, Xiangxiang Han, Yang Chen, Hao Chen, Zunhui Wan, Bing Song

**Affiliations:** ^1^The First Clinical Medical College of Lanzhou University, Lanzhou University, Lanzhou, China; ^2^Department of Cardiovascular Surgery, First Hospital of Lanzhou University, Lanzhou, China

**Keywords:** transcatheter aortic valve replacement, left bundle branch block, mortality, hospitalization for heart failure, permanent pacemaker implantation

## Abstract

**Background:**

Left bundle branch block (LBBB) is a common complication of the transcatheter aortic valve replacement (TAVR), and its impact on prognosis is controversial.

**Methods:**

A comprehensive electronic search was conducted in databases (PubMed, Embase, Cochrane Library, and The Web of Science), from the date of database establishment till March 2021, to screen for studies on new-onset LBBB after TAVR. We next performed a meta-analysis to evaluate the effect of new-onset LBBB after TAVR on patient prognosis, based on the Preferred Reporting Items for Systematic Reviews and Meta-Analyses (PRISMA) statement.

**Results:**

A total of 17 studies, including 9205 patients, were eligible for our analysis. Patients with new-onset LBBB had elevated all-cause mortality risk verses patients without new-onset LBBB, during all follow ups. The relevant data are as follows: 30-day (RR:1.71; 95%CI:1.27–2.29; *P* < 0.001), 1-year (RR:1.31; 95%CI:1.12–1.52; *P* < 0.001), and 2-year (RR:1.31; 95%CI:1.09–1.56; *P* = 0.003) follow ups. Likewise, new-onset LBBB patients also experienced increased cardiovascular mortality, compared to non-new-onset LBBB patients, but only in the 1-year follow up (RR:1.49; 95%CI:1.23–1.82; *P* < 0.001). Hospitalization for heart failure was dramatically elevated in patients with new-onset LBBB verses non-new-onset LBBB, in all follow ups. The relevant data are as follows: 30-day (RR:1.56; 95%CI:1.13–2.12; *P* = 0.007), 1-year (RR:1.35; 95%CI:1.08–1.68; *P* = 0.007), and 2-year (RR:1.49; 95%CI:1.21–1.84; *P* < 0.001). Similarly, new-onset LBBB patients had higher PPI risk than non-new-onset LBBB patients, in all follow ups. The relevant data are as follows: 30-day (RR:3.05; 95%CI:1.49–6.22; *P* = 0.002), 1-year (RR:2.15; 95%CI:1.52–3.03; *P* < 0.001), and 2-year (RR:2.52; 95%CI:1.68–3.78; *P* < 0.001).

**Conclusion:**

Patients with new-onset LBBB have worse prognosis after TAVR than those without new-onset LBBB. Recognition of the adverse effects of post-TAVR new-onset LBBB can lead to the development of new strategies that enhance clinical outcomes.

**Systematic Trial Registration:**

https://www.crd.york.ac.uk/prospero/display_record.php?RecordID=197224, identifier: 19722.

## Introduction

With increase in life expectancy and a growing aged population, aortic stenosis (AS) has become one of the most common valvular heart diseases as of today (1). In fact, the current prevalence of severe AS, among people >75 years of age, is 3.4% (2). When symptomatic, the 2-year mortality rate is observed in approximately 50% of severe AS cases (3). Because AS involves a mechanical obstruction, drug treatments are often ineffective, and valve replacement remains the only solution that can improve clinical symptoms and prolong life (4). Since its introduction in 2002, transcatheter aortic valve replacement (TAVR) has gradually emerged as an alternative to in the higher-risk population surgical aortic valve replacement (SAVR) procedure for patients with severe AS (5–7).

Fortunately, the development of valve implantation technology, the wide application of surgery, the accumulation of experience of the operators, and the emergence of new valve prosthesis have greatly reduced the incidence of serious complications (8). However, conduction disturbance after TAVR remains a highly common complication, mainly involving the new-onset left bundle branch block (LBBB) and the high-grade atrioventricular block (AVB), thereby requiring permanent pacemaker implantation (PPI), with a 4–30% incidence of the balloon-expandable valve and an 18–65% incidence of self-expanding valve (9). LBBB can lead to electromechanical asynchrony of the left ventricular activation, leading to ventricular pathological remodeling and left ventricular dysfunction. These pathological alterations can contribute to an elevated heart failure risk and poor prognosis (10, 11).

Till date, the prognostic effect of the new-onset LBBB after TAVR remains controversial. Conclusions from prior meta-analysis results were contradictory, and the studies were only conducted over 1 year of follow-up (12, 13). This may obscure the true long-term effect of new-onset LBBB. High quality meta-analysis is increasingly regarded as one of the key tools of obtaining evidence for clinical efficacy (14). Our aim, therefore, was to undertake a comprehensive and systematic overview of the clinical outcomes of patients, with and without new-onset LBBB following TAVR, during the 30-day, 1, and 2-year follow ups.

## Materials and Methods

This study was conducted, according to the PRISMA (Preferred Reporting Items for Systematic Reviews and Meta-Analyses) guidelines. The complete study protocol is registered in the PROSPERO international database (CRD42020197224) (15, 16). Additionally, the methodological quality was assessed with A Measurement Tool to Assess Systematic Reviews (17, 18).

### Search Strategy

The PubMed, Cochrane Library, Web of Science, and EMBASE databases were systematically searched from the establishment of the databases till the end of March 2021, using keywords “transcatheter aortic valve implantation,” “transcatheter aortic valve replacement,” “TAVI,” “TAVR,” “bundle-branch block,” “heart bundle branch block,” and “LBBB.” To ensure no relevant publications were overlooked, we also manually searched for qualifying publications in the reference lists of eligible articles.

### Inclusion and Exclusion Criteria

The authors screened the titles and study abstracts, based on the inclusion/exclusion criteria. Publications encompassing the following criteria were included in this meta-analysis: (1) AS patients who received TAVR; (2) contained new-onset LBBB incidence report; (3) examined the clinical outcomes of interest in a ≥ 1-year follow up study; and (4) consisted of non-new-onset LBBB controls. Among the publications excluded from the meta-analysis were conference reports, reviews, case reports, summaries, editorials, and studies published in a language other than English.

### Data Extraction

Based on the PRISMA statement, two authors extracted patient data, including first author, region, year, study type, number of patients, inclusion and exclusion criteria, gender, age, past history, echocardiography data, NYHA grade, logistic EuroSCORE, STS-PROM, valve type, access site, follow-up time, all-cause mortality, cardiovascular mortality, hospitalization for heart failure and PPI, and studied the data using standardized data extraction tables. To ensure accuracy, the data was further verified by a third author.

### Quality Assessment

The Newcastle Ottawa scale was employed for the assessment of eligible publication, and it primarily focused on the selection of study group, comparability between groups, and determination of exposure. We assigned a score of 0–9 for each study, following the evaluation. Higher scores represented higher study quality.

### Definitions and Outcomes

The primary outcome of this study was all-cause mortality during the 30-day, 1, and 2-year follow ups after TAVR. Secondary outcomes examined were cardiovascular mortality, hospitalization rate for heart failure, and PPI during the 30-day, 1, and 2-year follow ups post TAVR.

### Statistical Analysis

Continuous variables are represented by standardized mean and standard deviation, and categorical variables are expressed by percentages. We employed the random-effects model to compute risk ratio (RR) and 95% confidence interval (CI). We also used Cochran's Q statistic and *I*^2^ to test the heterogeneity across studies. When *P* < 0.10 or *I*^2^ ≥ 50%, the heterogeneity of the study was considered significant. The publication bias was visually evaluated using a funnel plot, and Egger's test was used to quantify publication bias. The robustness of the results and the effect of potential effect modifiers were examined with sensitivity analysis. All *P*-values were two-sided and P < 0.05 was considered statistically significant. Stata15.0 statistical analysis software was used for data analysis.

## Results

### Characteristics of Eligible Studies

**Figure 1:**
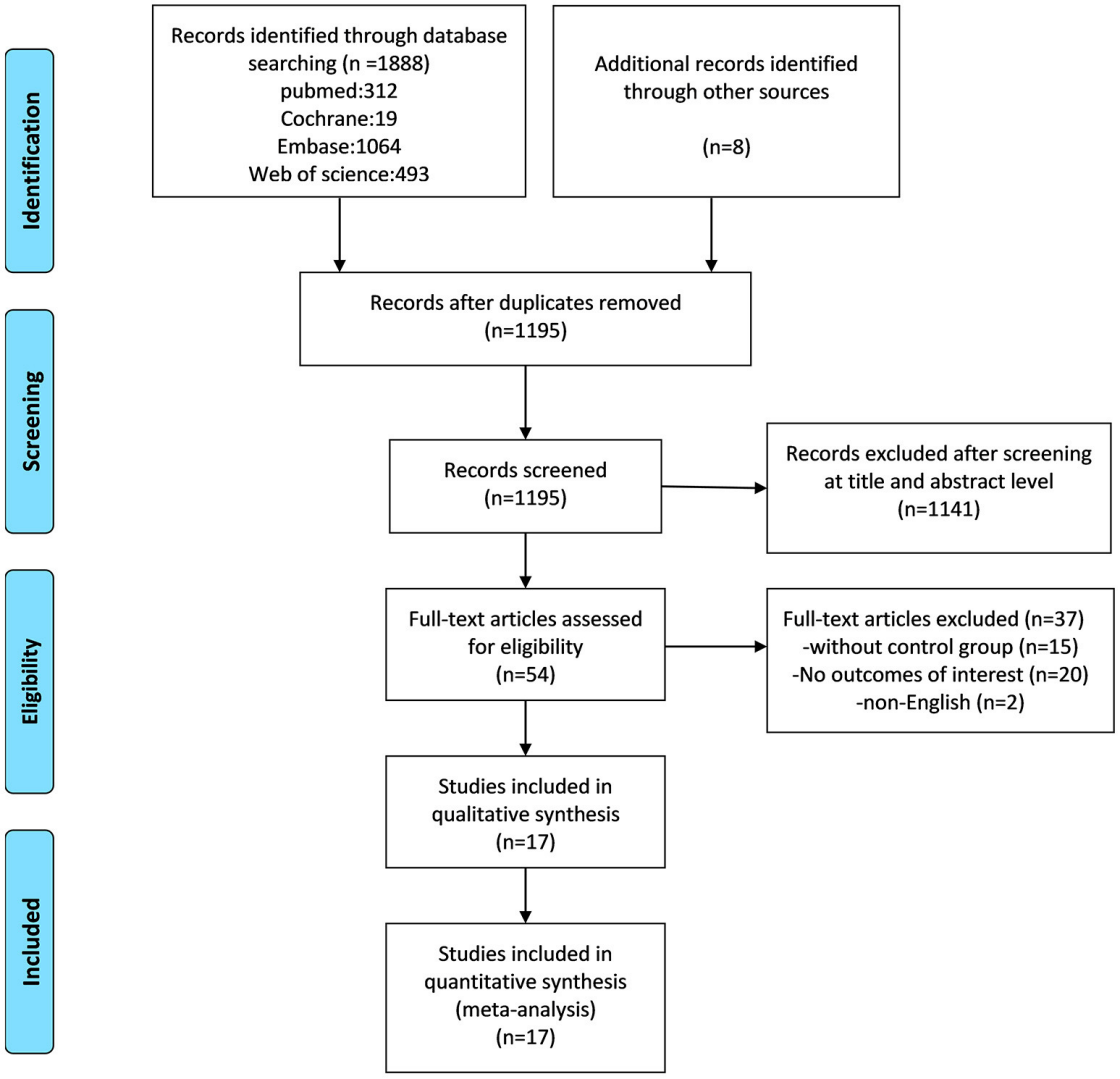
A flow diagram of the study selection process, based on the PRISMA statement.

As illustrated in Figure 1, a total of 1,888 articles were selected in the preliminary search. In addition, 8 suitable articles were obtained from the reference lists of the above-mentioned publications. Upon elimination of duplicate studies, 1,195 publications were screened by title and abstract. Among them, 54 were read in full to evaluate inclusion in the meta-analysis, 38 were eliminated, due to the following reasons: 15 publications were without a control group, 21 did not have an outcome of interest, and 2 were non-English. Finally, 9,205 patients, in 17 publications (19–35), met the inclusion criteria for the final meta-analysis. Details of the updated eligible publications are reported in Table 1.

**Table 1:** Characteristics of included studies.

**Reference**	**Country**	**Enrolment years**	**New-onset LBBB definition**	**Incidence of LBBB**	**LBBB**	**No LBBB**	**Type of valve**	**NOS**
Houthuizen et al. (19)	Netherlands	2005–2010	Postprocedural	32.9%	233	446	BEV and SEV	8
Franzoni et al. (20)	Italy	2007–2011	Postprocedural	26.5%	63	175	BEV and SEV	7
Testa et al. (21)	Italy	2007–2011	at discharge	22.5%	224	594	SEV	9
Houthuizen et al. (22)	International	2006–2011	Persistent 12-month after TAVR	23.3%	111	365	BEV and SEV	8
Nazif et al. (23)	International	2007–2009	At discharge or within 7 days of procedure	10.5%	121	1,030	BEV	9
Schymik et al. (25)	Germany	2008–2012	At discharge	31.1%	197	437	BEV and SEV	8
Urena et al. (32)	Canada, Spain	NR	At discharge or if LBBB and die before discharge	19.2%	79	589	BEV	9
Carrabba et al. (24)	Italy	NR	At discharge	37%	34	58	BEV and SEV	8
Lopez-Aguilera et al. (26)	Spain	2008–2014	At discharge	52%	80	73	SEV	9
Kessler et al. (30)	Germany	2014–2016	At discharge	Not applicable	264	264	BEV, SEV and MEV	9
Chamandi et al. (27)	International	2007–2015	At discharge or if LBBB and die before discharge	20.1%	212	808	BEV, SEV and MEV	9
Eschalier et al. (28)	France	2015–2017	Postprocedural persisting for more than 24 hours	Not applicable	40	40	BEV and SEV	7
Nazif et al. (31)	International	2011–2014	At discharge	15.2%	179	1,000	BEV	9
Jorgensen et al. (29)	Denmark	2007–2017	Persistent LBBB without PPI within 30 days after TAVR	34.7%	237	447	BEV and SEV	8
Sasaki et al. (33)	Japanese	2016–2018	Postprocedural	12.6%	29	201	BEV and SEV	8
Hamandi et al. (35)	USA	2012–2016	At discharge	12.3%	52	372	BEV and SEV	7
Akdemir et al. (34)	USA	2012–2015	At discharge	31.1%	47	104	BEV, SEV and MEV	8

*PPI, permanent pacemaker implantation; LBBB, left bundle-branch block; BEV, balloon-expandable valve; SEV, self-expanding valve; MEV, mechanically-expandable valve; NOS, Newcastle-Ottawa Scale; NR, not reported*.

### Baseline Characteristics of the Eligible Cohorts

**Figure 2:**
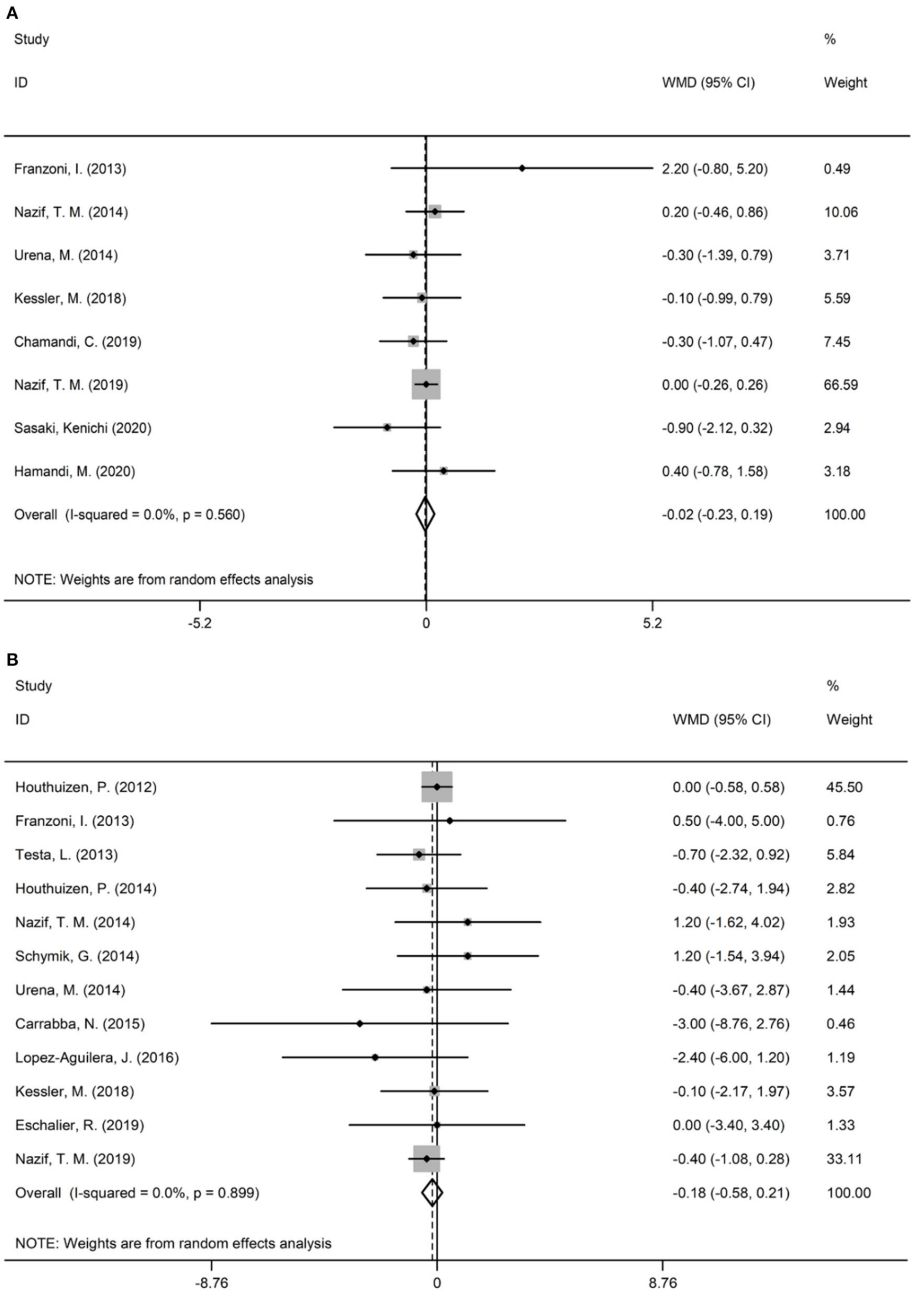
Forest plots illustrating the surgical risk score. **(A)** STS score; **(B)** Logistic EuroSCORE.

Table 2 summarizes the baseline and procedural characteristics of patients included in the selected publications. There were no significant differences in the STS score and logistic EuroSCORE between the two groups (Figure 2). Generally, the most commonly used vascular access is the TF-TAVR approach. Among all studies, three employed balloon-expandable valves, three others employed self-expanding valves, and the remaining employed multiple categories of valves.

**Table 2:** Patient and procedural characteristics of included studies.

**Study**	**Male**	**Age, y**	**STS score**	**Logistic EuroSCORE**	**LVEF**	**DM**	**HTN**	**NYHA III or IV**	**CAD**	**COPD**	**Transfemoral**
Houthuizen et al.	44/48	82 ± 2/ 81 ± 2	NR	16 ± 4/16 ± 4	NR	28/21	NR	NR	48/46	26/26	NR
Franzoni et al.	48/56	79 ± 7/80 ± 7	10 ± 11/8 ± 7	23 ± 16/22 ± 15	52 ± 12/53 ± 12	35/25	83/73	67/64	51/35	NR	NR
Testa et al.	42/47	82 ± 5/ 82 ± 7	NR	23 ± 10/24 ± 12	53 ± 11/51 ± 13	25/24	NR	72/76	NR	NR	90/88
Houthuizen et al.	57/40	80 ± 5/81 ± 7	NR	16 ± 11/16 ± 11	NR	81/10	NR	81/81	52/53	33/26	82/58
Nazif et al.	43/44	84 ± 7/84 ± 7	11 ± 4/11 ± 4	26 ± 15/25 ± 16	54 ± 11/55 ± 12	45/37	94/92	93/96	80/75	42/45	50/57
Schymik et al.	33/41	82 ± 6/82 ± 6	NR	23 ± 17/21 ± 16	59 ± 13/59 ± 13	37/32	NR	NR	59/55	11/13	NR
Urena et al.	49/49	78 ± 9/81 ± 8	8 ± 5/8 ± 5	21 ± 14/21 ± 14	56 ± 11/56 ± 13	44/29	90/80	79/77	73/68	28/27	38/57
Carrabba et al.	53/52	81 ± 6/81 ± 7	NR	18 ± 12/21 ± 16	48 ± 12/48 ± 15	41/24	56/62	NR	24/31	21/17	NR
Lopez-Aguilera et al.	45/59	78 ± 5/77 ± 6	10 ± 11/12 ± 11	15 ± 9/18 ± 13	58 ± 15/56 ± 13	26/26	63/47	NR	28/25	84/71	NR
Kessler et al.	46/46	81 ± 6/80 ± 6	7 ± 5/7 ± 5	13 ± 13/13 ± 12	59 ± 14/57 ± 16	27/30	NR	NR	61/58	63/62	100
Chamandi et al.	56/57	80 ± 7/81 ± 8	7 ± 5/7 ± 5	NR	60 ± 13/56 ± 13	39/34	NR	78/69	40/42	33/28	92/82
Eschalier et al.	53/63	82 ± 5/82 ± 5	NR	13 ± 9/13 ± 7	NR	33/30	93/70	30/40	60/50	15/15	80/73
Nazif et al.	53/54	81 ± 7/82 ± 7	6 ± 2/6 ± 2	5 ± 4/6 ± 5	60 ± 10/59 ± 11	45/36	94/93	81/75	72/75	32/31	84/83
Jorgensen et al.	53/50	81 ± 3/81 ± 2	NR	NR	55 ± 3/55 ± 4	22/20	81/77	67/63	52/50	24/21	98/89
Sasaki et al.	24/37	84 ± 5/84 ± 6	5 ± 3/6 ± 6	NR	64 ± 12/64 ± 10	28/24	83/80	17/19	NR	10/11	NR
Hamandi et al.	42/53	83 ± 7/81 ± 9	8 ± 4/7 ± 4	NR	57 ± 9/54 ± 13	50/45	98/94	83/82	NR	25/22	94/83
Akdemir et al.	53/46	80 ± 11/79 ± 9	NR	NR	53 ± 11/56 ± 11	36/28	81/95	NR	83/72	28/29	100

*Data were given as mean ± SD, n, or n (%). CAD, coronary artery disease; HTN, hypertension; LVEF, left ventricular ejection fraction; EuroSCORE, European system for cardiac operative risk evaluation; STS, the Society of Thoracic Surgeons risk score; COPD, chronic obstructive pulmonary disease; DM, diabetes mellitus; NR, not reported*.

### Assessment of Risk of Bias

Based on the Newcastle Ottawa scale (Table 1), the quality of the selected publications was generally high. No evidence of publication bias was found, as evidenced by the funnel plot and Egger's test.

### All-Cause Mortality

**Figure 3:**
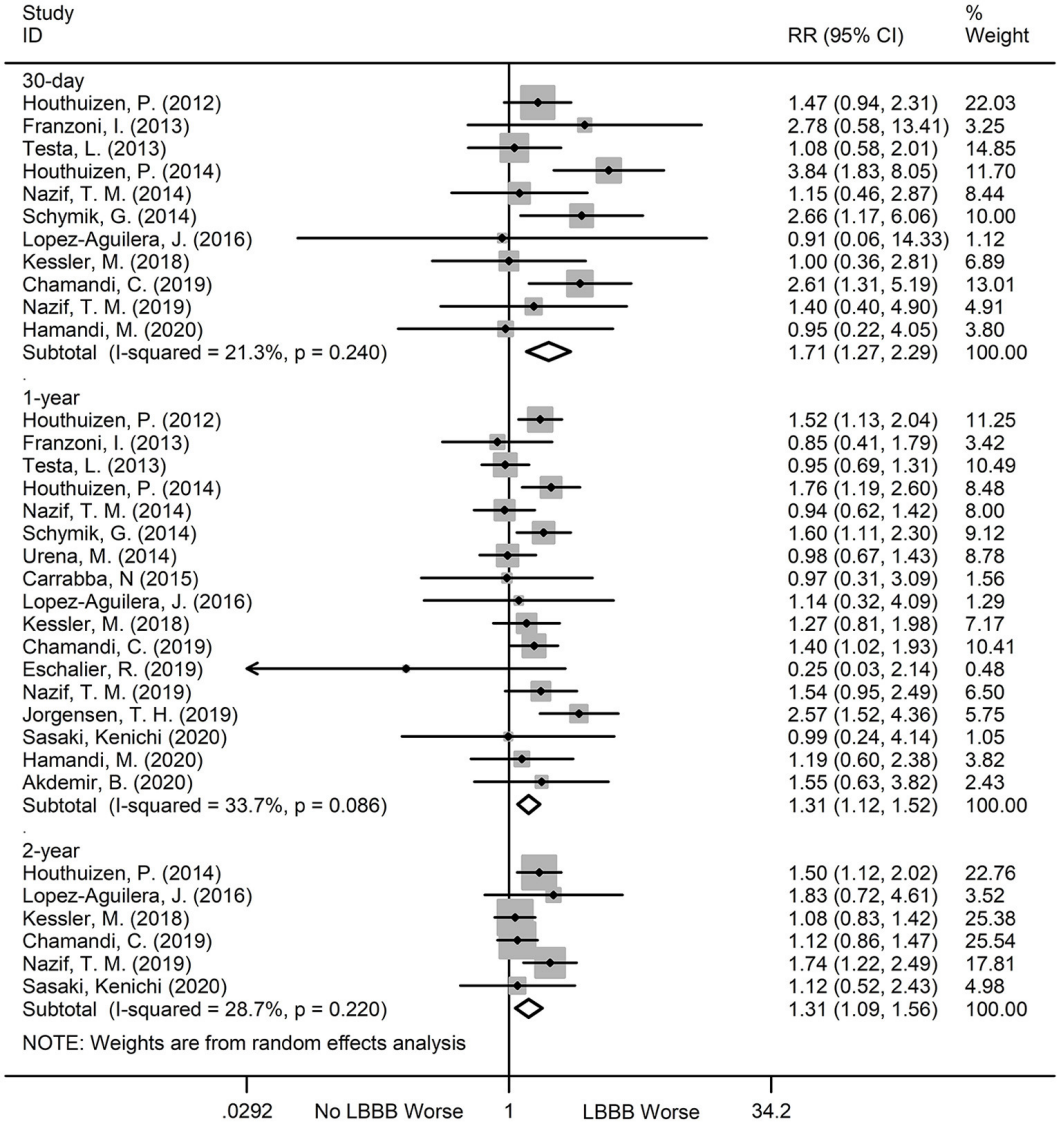
Forest plot comparing all-cause mortality risk between patients with and without new-onset LBBB after TAVR.

All-cause mortality was the primary outcome of our meta-analysis. At the 30-day follow up, 11 publications reported an association between new-onset LBBB and all-cause mortality. In particular, patients with new-onset LBBB after TAVR had a higher risk of all-cause mortality than those without new-onset LBBB (RR:1.71; 95%CI:1.27–2.29; *P* < 0.001; *I*^2^ = 21.3%). At the 1-year follow up, 17 publications reported all-cause mortality, and the patients with new-onset LBBB had a higher all-cause mortality (RR:1.31; 95%CI:1.12–1.52; *P* < 0.001; *I*^2^ = 33.7%) relative to those without. Finally, at the 2-year follow up, 6 publications reported all-cause mortality, with an increased risk of all-cause mortality among the new-onset LBBB patients vs. non-new-onset LBBB patients after TAVR (RR:1.31; 95%CI:1.09–1.56; *P* < 0.001; *I*^2^ = 28.7%) (Figure 3). No significant heterogeneity was observed among the publications. Additionally, sensitivity analysis, performed by one by one exclusion study, failed to alter the conclusion of our analysis.

### Cardiovascular Mortality

**Figure 4:**
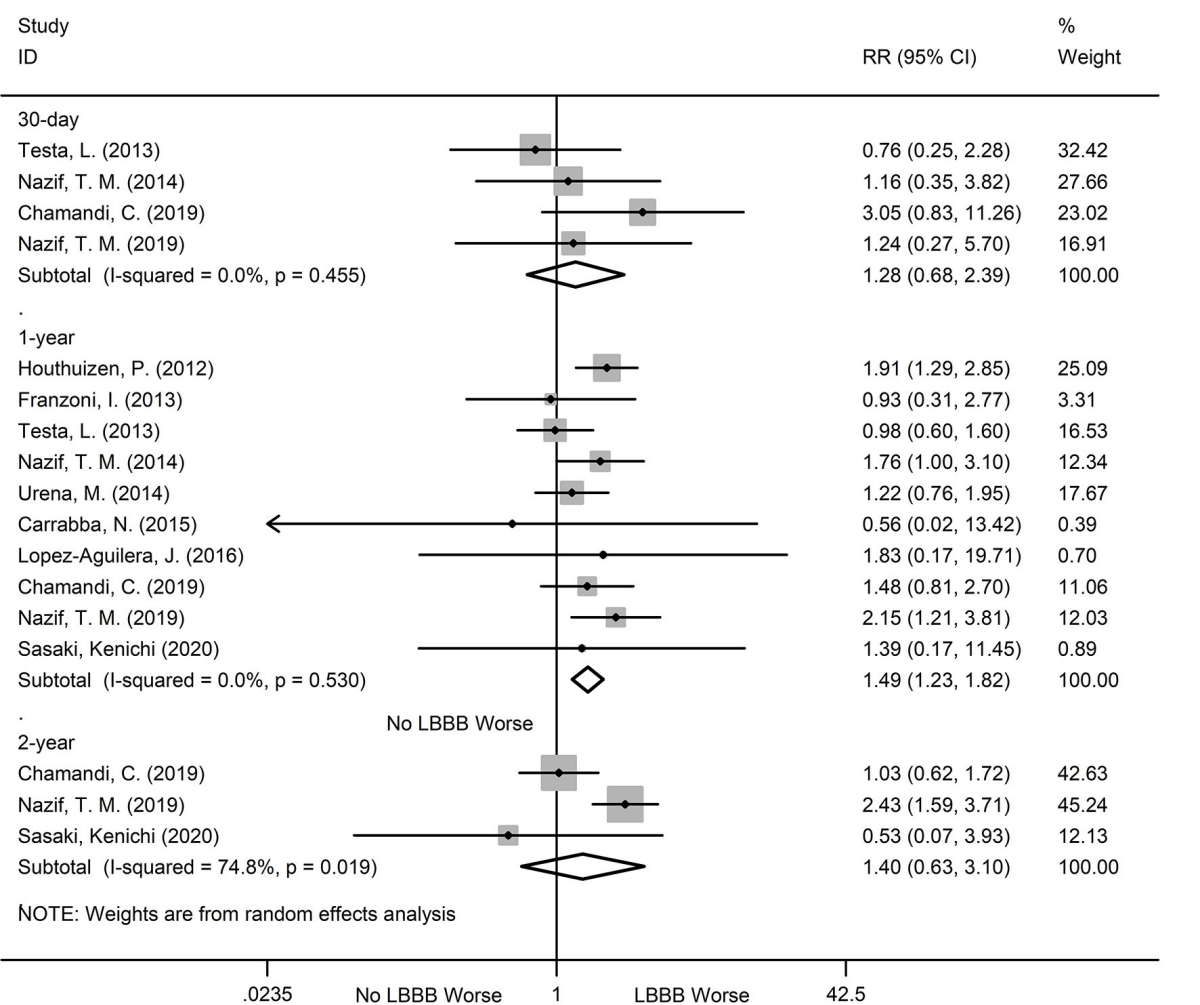
Forest plot comparing cardiovascular mortality risk between patients with and without new-onset LBBB after TAVR.

There was no statistically significant difference in the 30-day (RR:1.28; 95%CI:0.68–2.39; *P* = 0.445; *I*^2^ = 0%) and 2-year (RR:1.40; 95%CI:0.63–3.10; *P* = 0.404; *I*^2^ = 74.8%) cardiovascular mortality between patients with new-onset LBBB and those without. However, the new-onset LBBB was associated with significantly higher 1-year cardiovascular mortality (RR:1.49; 95%CI:1.23–1.82; *P* < 0.001; *I*^2^ = 0%), compared to the non-new-onset LBBB patients (Figure 4). We observed significant heterogeneity (*I*^2^ = 74.8%) at the 2-year follow-up. However, after excluding the studies one by one, the results of sensitivity analysis remained the same.

### Hospitalization for Heart Failure

**Figure 5:**
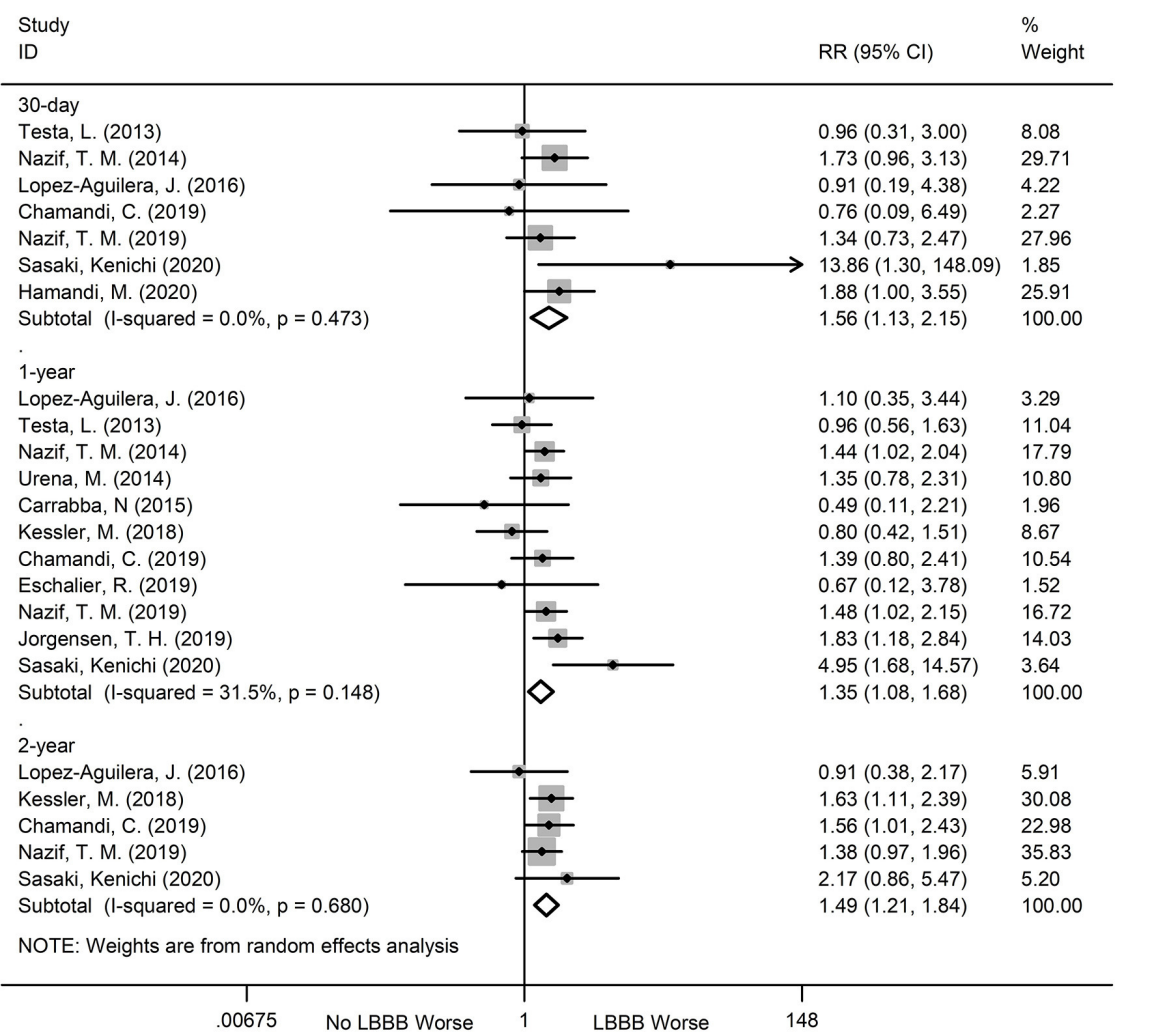
Forest plot comparing hospitalization for heart failure risk between patients with andwithout new-onset LBBB after TAVR.

During the 30-day, 1, and 2-year follow ups, the new-onset LBBB patients after TAVR showed a dramatic increase in hospitalization risk for heart failure, relative to non- new-onset LBBB patients. The relevant data are as follows: 30-day (RR:1.56; 95%CI:1.13–2.15; *P* = 0.007; *I*^2^ = 0%), 1-year (RR:1.35; 95%CI:1.08–1.68; *P* = 0.007, *I*^2^ = 31.5%), 2-year (RR:1.49; 95%CI:1.21–1.84; *P* < 0.001; *I*^2^ = 0%) (Figure 5). Additionally, no obvious heterogeneity was observed, and there was no obvious change in sensitivity analysis.

### PPI

**Figure 6:**
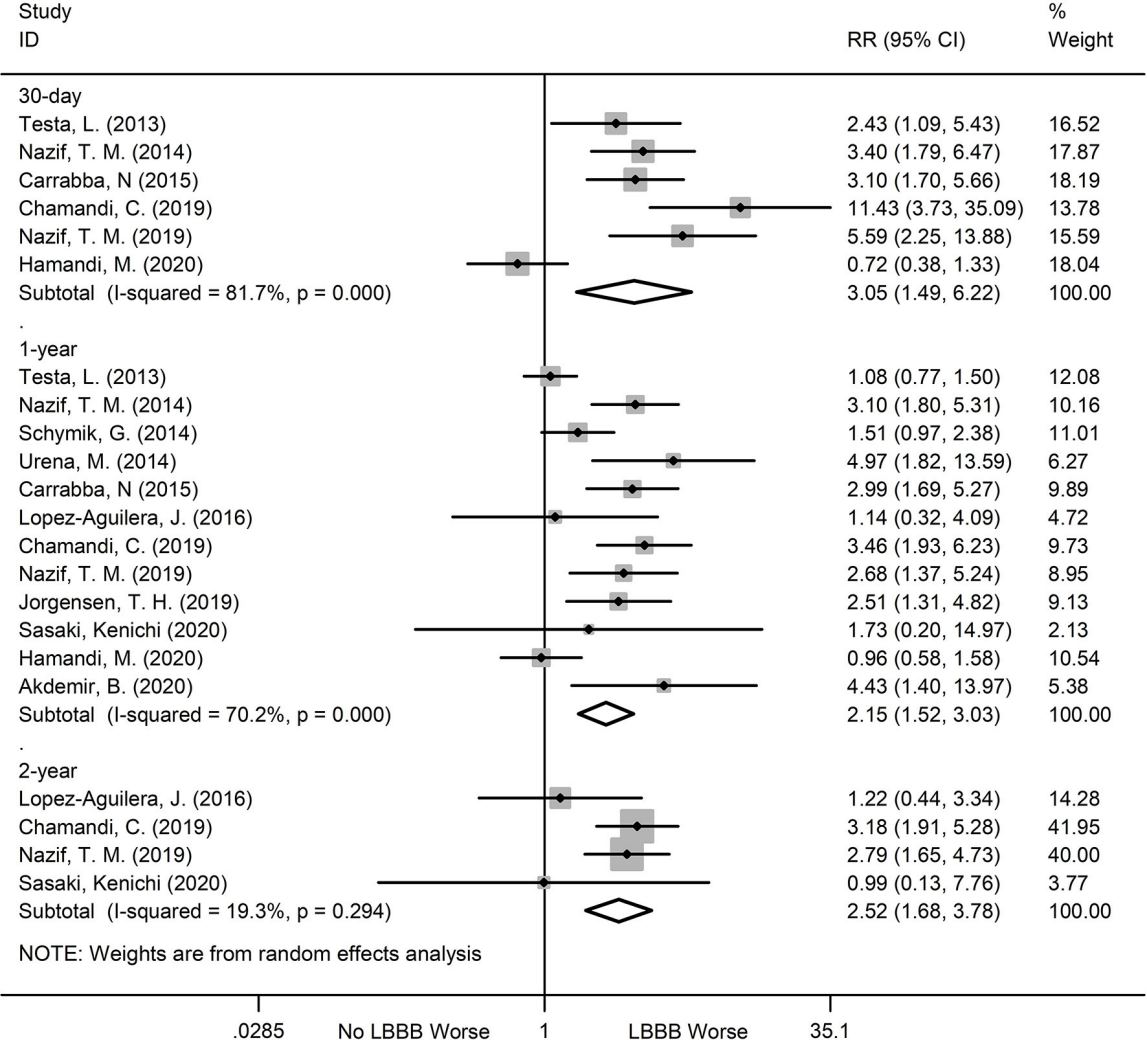
Forest plot comparing PPI risk between patients with and without new-onset LBBB after TAVR.

The new-onset LBBB patients after TAVR were also more likely to undergo PPI, during all follow ups, compared to the non-new-onset LBBB patients. The relevant data are as follows: 30-day (RR:3.05; 95%CI:1.49–6.22; *P* = 0.002; *I*^2^ = 81.7%), 1-year (RR:2.15;95%CI:1.52–3.03; *P* < 0.001; *I*^2^ = 70.2%), 2-year (RR:2.52; 95%CI:1.68–3.78; *P* < 0.001; *I*^2^ = 19.3%) (Figure 6). Significant heterogeneity was observed among the two groups (*I*^2^ = 81.7% and *I*^2^ = 70.2%), but the sensitivity analysis did not markedly alter the results.

## Discussion

Till date, our meta-analysis and systematic review offers the greatest relationship between new-onset LBBB and patient prognosis, and is the first to conduct pooled analyses of long-term prognosis of new-onset LBBB after TAVR. Our analysis demonstrated 2 main findings: (1) patients with new-onset LBBB, but not without new-onset LBBB, experience an increased risk of all-cause mortality, hospitalization for heart failure, and PPI at the 30-day, 1 and 2-year follow ups post TAVR; and (2) patients with new-onset LBBB also have an increased risk of cardiovascular mortality 1 year after TAVR, as opposed to those without new-onset LBBB.

LBBB is generally considered to be a marker of poor prognosis (10, 36). Our analysis revealed that patients with new-onset LBBB had a higher risk of all-cause mortality than those without new-onset LBBB. The reason may be multifactorial. On one hand, the adverse prognosis may be related to the ventricular asynchrony, diastolic shortening, and ventricular septal motion abnormality, caused by LBBB itself. With the progression of time, this can lead to a reduction in LVEF, resulting in asymmetric dilation, hypertrophy of the heart, and ultimately heart failure (10). Additionally, LBBB may also increase the risk of life-threatening ventricular arrhythmias, severe bradyarrhythmias, and sudden death (33). Previous studies investigating the effects of new-onset LBBB after TAVR on patient prognosis provided inconsistent results. For example, the meta-analysis by Regueiro et al. (12). did not show an association between new-onset LBBB and all-cause mortality (RR:1.21; 95%CI:0.98–1.50). However, a strong positive association between new-onset LBBB and all-cause mortality risk was reported (RR:1.32; 95%CI:1.17–1.49) in another study (13). Similarly, several retrospective investigations reported that new-onset LBBB after TAVR has no effect on long-term mortality (26, 27, 30), while other studies suggested that LBBB is a marker of enhanced long-term mortality after TAVR (22, 31). This obvious discrepancy in conclusions may be due to the difference in LBBB patient populations and insufficient follow-up time. To circumvent these limitations, our meta-analysis was the first to include a 2-year follow-up and demonstrated that LBBB after TAVR was, in fact, associated with significant increases in all-cause mortality, hospitalization for heart failure and PPI during all follow ups, and cardiovascular mortality, only at the 1-year follow up.

The effect of new-onset LBBB after TAVR on heart failure-driven hospitalization is also controversial. Some studies reported no association between new-onset LBBB and increased risk of hospitalization for heart failure after TAVR (21, 32). Conversely, in a meta-analysis involving pooled data from six studies, Faroux et al. (13). revealed that new-onset LBBB significantly increased the incidence of 1-year hospitalization for heart failure (RR1.35, 95%CI 1.05–1.72). Using extensive overview of published reports, we compiled available evidence on this issue, that revealed that the new-onset LBBB after TAVR does, in fact, increase the rate of hospitalization for heart failure. This may be related to the ventricular remodeling and left ventricular functional deterioration, caused by LBBB. During the 6–12 months follow-ups, Nazif et al. (23) showed that LVEF failed to improve after TAVR in the newly-onset LBBB patients and the values remained lower than that in patients without LBBB (53 vs. 58%, *p* < 0.001). Moreover, Carabba et al. (24). demonstrated that in patients without conduction disorders, LVEF was significantly improved in the early stage after TAVR and gradually stabilized over time. However, the recovery of cardiac function was slow in patients with new-onset LBBB after TAVR, as opposed to patients without new-onset LBBB, and LVEF showed a downward trend at the 1-year follow-up.

Our study also demonstrated an increased risk of PPI in the new-onset LBBB patients after TAVR, relative to non-new-onset LBBB patients. This is consistent with other studies confirming the effects of LBBB on PPI after TAVR. Data from two previous meta-analyses confirmed a two-fold increase in the risk of 1-year PPI in new-onset LBBB patients after TAVR, relative to non-new-onset LBBB patients (12, 13). From an electrophysiological point of view, the risk of LBBB progression to complete AVB after TAVR is relatively high, due to the near complete disruption of the left bundle branch conduction in these patients, even though the right bundle branch remains sufficiently activated (33). Furthermore, TAVR is mostly implanted in elderly patients, who have a high prevalence of conduction system dysfunctions, along with an increased tendency to develop high AVB from LBBB (37). Interestingly, in our meta-analysis, we discovered that patients with new-onset LBBB had the highest risk of early PPI, as opposed to non-new-onset LBBB patients. This is likely due to the fact that doctors have a lower diagnostic threshold, brought on by fear of progressing to high AVB (23). Moreover, postoperative septal inflammation, compression, and edema can increase the risk of early progression to high AVB, while over time, compression and inflammation resolution can reduce the risk of high AVB and therefore, reduce the demand for PPI (34).

Interestingly, the LBBB incidence in first-generation valvular devices after TAVR is much higher (4–65%) than with SAVR (2.3–8.6%) (38, 39). Possible reasons could include usage of different surgical equipment, records of only transient or persistent LBBB, differences in the risk of conduction disturbances, and different time points of ECG collection (40). New-onset LBBB with the SAPIEN 3 valve ranges from 6 to 29% and appears to be similar to the prior generation valve, while New-onset LBBB rate with the CoreValve Evolut R system seems to be lower than that reported with the prior CoreValve system (9). The use of mechanically expandable lotus valves confers the largest LBBB incidence after TAVR (55–77%) (41–43). Most of the conduction disturbances (90%) occur during the first week post valve implantation (44). New-onset LBBB, on the other hand, can be transient, for instance, in 19–34% of patients, it can disappear within the first few days, but in most patients (62%), it can still be detected on the 30-day follow up (21). However, it can also become a long-lasting condition, as is seen in about 66% of patients who suffer from new-onset LBBB for a year or the 0.8% of patients who experience new-onset LBBB >1-year after TAVR (37). Compared to the Edwards system, LBBB spontaneous recovery was much less frequent than with the CoreValve system (39 vs. 9.5%) (22). LBBB is an anatomic condition that occurs due to the proximity of the aortic annulus to the atrioventricular nodal-Hisian conduction system, which allows the conduction tissue in the ventricle to be vulnerable to damage, during TAVR (45). Additionally, continuous radial force, transient tissue inflammation, edema, and ischemia are thought to contribute to the possible mechanisms of abnormal conduction after TAVR (46). Multiple studies have established that predictors like valve implantation depth (47), mean aortic gradient (34), degree of annular calcification (48), prosthesis type (25), and pre-existing right bundle branch block (49), can play an important role in post-TAVR conduction abnormalities.

Due to its long-term safety implications, TAVR is more commonly used for low-to-intermediate risk patients. At present, the management of LBBB after TAVR has not been clearly defined by an international standard, so each treatment center has developed its own management strategies. Hence, it is necessary to improve identification of the predictive biomarkers for LBBB, as prompt post-procedural identification and treatment of new-onset LBBB can significantly reduce post-procedural complications. TAVR-induced LBBB is often related to a decrease in global longitudinal and radial systolic function. Therefore, prompt cardiac resynchronization therapy may restore inter-and intra-ventricular dyssynchrony and may be effective in improving left ventricular function and reducing rehospitalization incidence for heart failure. Moreover, given the adverse effects of LBBB on ventricular remodeling, operators need to take extra precaution to reduce the risk of LBBB induced by TAVR. A recent study showed that preprocedural CT imaging can help identify risk factors for conduction disturbances, such as membranous septum length, device landing zone calcium, and annular size (50). The Minimizing Depth According to the Membranous Septum (MIDAS) approach, in which operators attempted to position the self-expandable valve at a prerelease depth in relation to the non-coronary cusp of length smaller than that of the membranous septum, significantly reduced the rate of new PPI (3.0 vs. 9.7%) and new-onset LBBB (9.0 vs. 25.8%) without valve embolization or a need of second valve (51). Periprocedural planning based on the CT findings is important to reduce the risk of conduction disturbances after TAVR. Lastly, to reduce the occurrence of LBBB and prevent the potential impact of LBBB on patient prognosis, the following steps are encouraged: strictly screen patients before surgery; mastery of indications; selection of optimal surgical approach and matching valve system.

## Limitations

Our meta-analysis had several potential limitations: (1) we mostly analyzed observational studies, which may have introduced bias; (2) we may have unintentionally introduced information bias, due to the limited availability of original data; moreover, we extracted some RR mortality data from the Kaplan Meier curve, which may have reduced the accuracy of our results; (3) our analysis of the mortality and PPI risk in the new-onset LBBB patients post-TAVR showed heterogeneity. However, the sensitivity analysis revealed no change; (4) some of the publications, in our analysis, had non-uniformity; (5) the publication selection process was limited to the English language, which may have introduced potential language bias.

## Conclusions

Based on our meta-analysis, patients with new-onset LBBB after TAVR had a higher risk of all-cause mortality, hospitalization for heart failure, and PPI, compared to those without new-onset LBBB. With TAVR indications expanding to patients with low surgical risk, conduction block remains an ongoing problem, and future efforts must be undertaken to identify factors associated with the progression of conduction disturbances and strengthen management to improve patient clinical outcomes.

## Data Availability Statement

The original contributions presented in the study are included in the article/supplementary material, further inquiries can be directed to the corresponding author.

## Author Contributions

Data analysis, interpretation, and manuscript writing were performed by JW. Literature search, study selection, data extraction, and quality assessment were performed by XH, YC, HC, and ZW. SL and BS were responsible for the conception, design of the study, and revised the manuscript carefully. All authors contributed to the article and approved the submitted version.

## Funding

This systematic review was supported by the Natural Science Foundation of Gansu Province (Grant No. 20JR10RA689).

## Conflict of Interest

The authors declare that the research was conducted in the absence of any commercial or financial relationships that could be construed as a potential conflict of interest.

## Publisher's Note

All claims expressed in this article are solely those of the authors and do not necessarily represent those of their affiliated organizations, or those of the publisher, the editors and the reviewers. Any product that may be evaluated in this article, or claim that may be made by its manufacturer, is not guaranteed or endorsed by the publisher.
